# Comprehensive profiling of immune-related genes in soft tissue sarcoma patients

**DOI:** 10.1186/s12967-020-02512-8

**Published:** 2020-09-01

**Authors:** Chuan Hu, Bo Chen, Zhangheng Huang, Chuan Liu, Lin Ye, Cailin Wang, Yuexin Tong, Jiaxin Yang, Chengliang Zhao

**Affiliations:** 1 Department of Orthopedic, Affiliated Hospital of Chengde Medical University, Hebei, China; 2 Qingdao University Medical College, Shandong, 266071 China; 3 Wenzhou Medical University, Zhejiang, 325000 China; 4 Department of Medical Oncology, The First Hospital of China Medical University, Shenyang, 110001 China

**Keywords:** Immune-related genes, Soft tissue sarcoma, Immune cell, Immune checkpoint, Prognosis, Transcription factor

## Abstract

**Background:**

Immune-related genes (IRGs) have been confirmed to have an important role in tumorigenesis and tumor microenvironment formation. Nevertheless, a systematic analysis of IRGs and their clinical significance in soft tissue sarcoma (STS) patients is lacking.

**Methods:**

Gene expression files from The Cancer Genome Atlas (TCGA) database and Genotype-Tissue Expression (GTEx) were used to select differentially expressed genes (DEGs). Differentially expressed immune-related genes (DEIRGs) were determined by matching the DEG and ImmPort gene sets, which were evaluated by functional enrichment analysis. Unsupervised clustering of the identified DEIRGs was conducted, and associations with prognosis, the tumor microenvironment (TME), immune checkpoints, and immune cells were analyzed simultaneously. Two prognostic signatures, one for overall survival (OS) and one for progression free survival (PFS), were established and validated in an independent set. Finally, two transcription factor (TF)-IRG regulatory networks were constructed, and a crucial regulatory axis was validated.

**Results:**

In total, 364 DEIRGs and four clusters were identified. OS, TME scores, five immune checkpoints, and 12 types of immune cells were found to be significantly different among the four clusters. The two prognostic signatures incorporating 20 DEIRGs showed favorable discrimination and were successfully validated. Two nomograms combining signature and clinical variables were generated. The C-indexes were 0.879 (95%CI 0.832 ~ 0.926) and 0.825 (95%CI 0.776 ~ 0.874) for the OS and PFS signatures, respectively. Finally, TF-IRG regulatory networks were established, and the MYH11-ADM regulatory axis was verified in three independent datasets.

**Conclusion:**

This comprehensive analysis of the IRG landscape in soft tissue sarcoma revealed novel IRGs related to carcinogenesis and the immune microenvironment. These findings have implications for prognosis and therapeutic responses, which reveal novel potential prognostic biomarkers, promote precision medicine, and provide potential novel targets for immunotherapy.

## Background

Soft tissue sarcomas (STSs) are a rare group of heterogeneous malignant tumors originating from mesenchymal tissue and comprise more than 50 histological subtypes [1, 2]. Although STSs only account for 1% of all malignancies, they account for approximately 10% of malignancies in children and adolescents [3, 4]. According to previous investigations, the total STS incidence is 2.49 – 5.87 per 100,000 person-years, and the 5-year survival rate after diagnosis is 55.5% –56.5% [5–7]. However, for advanced STS patients, the 5-year survival rate dramatically decreases to 27.2% [5]. In addition, 40% – 50% of STS patients develop distant metastases [8], which makes it difficult to select the most appropriate treatment, such as surgery, chemotherapy, or radiotherapy. Therefore, it is crucial to find accurate biomarkers for assessing risk in STS patients.

In recent years, several prognostic signatures based on lncRNA, miRNA, and plasmacytoma variant translocation 1 have been established for STS [9–11]. Nevertheless, these markers have been unable to be translated into clinical practice due to their poor prognostic ability and lack of validation. The role of immune-related features in malignancies has been a recent area of active research. Elements of the immune system have proven to be strong factors for tumorigenesis and tumor progression [12]. More importantly, previous studies have indicated that immune-related genes (IRGs) can serve as effective prognostic biomarkers of many tumors, such as lung cancer [13, 14], ovarian cancer [15], hepatocellular carcinoma [16], head and neck squamous cell carcinoma [17], papillary thyroid cancer [18], bladder urothelial carcinoma [19], and renal cancer [20]. However, the prognostic significance of IRGs in STS remains unclear.

Here, we performed a systematic analysis of IRGs in STS and determined STS-related IRGs. The potential function and underlying regulatory mechanisms of these IRGs were also investigated. Furthermore, the integration of clinicopathological data and RNA-sequencing data provides novel insights into the prognostic value of IRGs. Finally, we discerned distinct clusters of STSs based on IRGs and investigated the association between IRG-based clusters and immune checkpoints, the tumor microenvironment (TME), and immune cells. The present study reveals a complex immune landscape consisting of both a continuous spectrum and discrete clusters across STS patients.

## Methods

### Patients and datasets

**Fig. 1:**
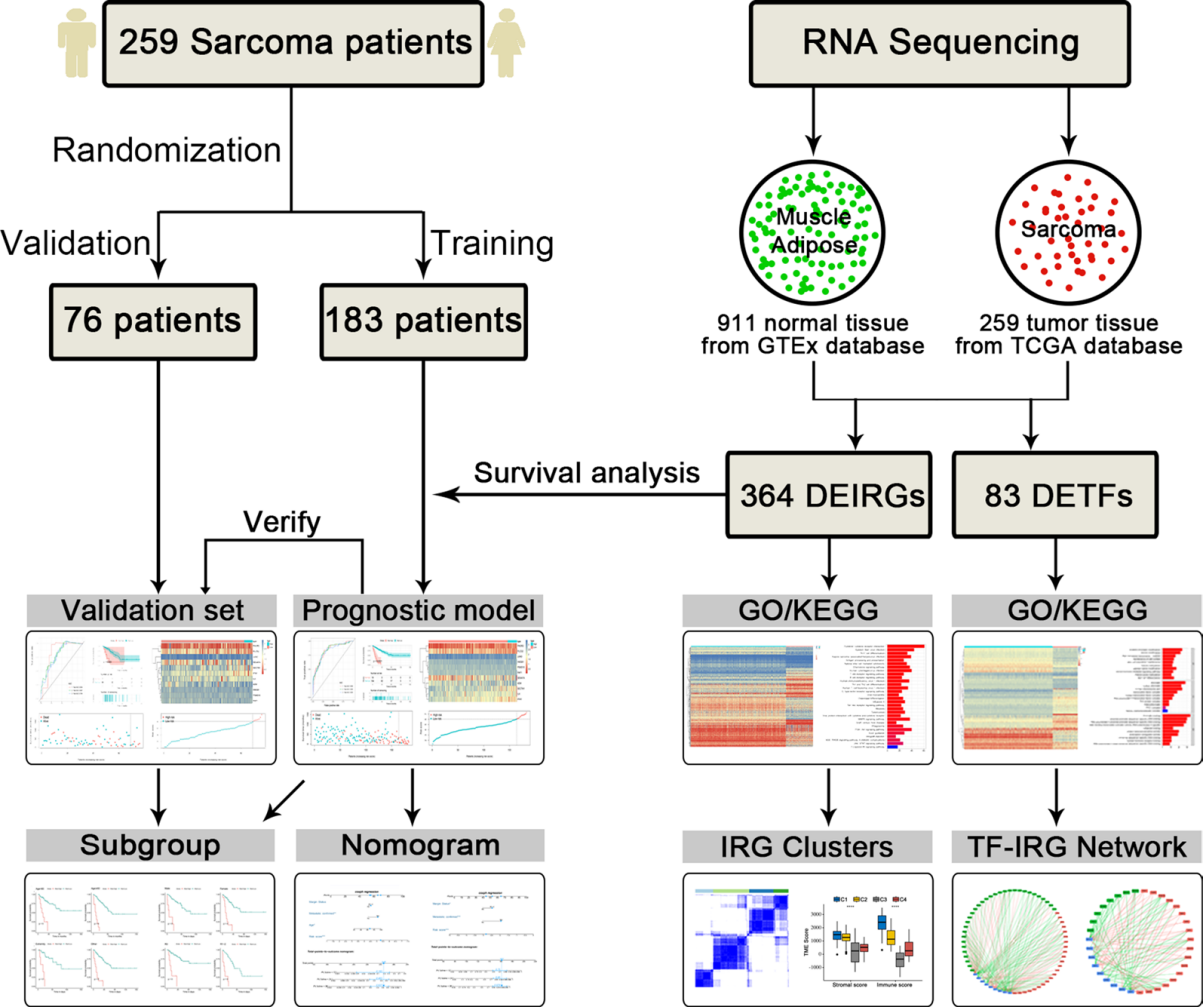
Flowchart of our study. 259 sarcoma patient samples and 911 normal muscle and adipose tissue samples were included in our study. 183 patients were incorporated into the training set and the remaining 76 patients were used to form the validation set. Comprehensive profiling of immune-related genes showed favorable prognostic value and demonstrated their significant association with immune features

RNA sequencing data from The Cancer Genome Atlas (TCGA) and Genotype-Tissue Expression (GTEx) datasets were downloaded from the UCSC Xena browser (https://xenabrowser.net/) [21]. TCGA is a publicly funded project that aims to catalog and discover major cancer-causing genome alterations in large cohorts of over 30 human tumors through large-scale genome sequencing and integrated multidimensional analyses. The GTEx project provides RNA sequencing data from samples that were collected from 54 nondiseased tissue sites across nearly 1000 individuals. For the GTEx and TCGA datasets, RNA sequencing data (FPKM values) were normalized into log_2_ (FPKM + 1). Meanwhile, the corresponding TCGA clinical data were downloaded from cBioPortal (http://www.cbioportal.org/) [22]. A total of 259 patients with STS were included, including 104 with leiomyosarcomas, 58 with dedifferentiated liposarcomas, 51 with undifferentiated pleomorphic sarcomas, 25 with myxofibrosarcomas, 10 with synovial sarcomas, and 11 with other STS types. 4The clinicopathological characteristics of the patients enrolled in our study are shown in Table 1. Additionally, the entire set of 2498 IRGs (Additional file 1) was collected from the ImmPort database (https://www.immport.org/shared/) [23]. A flowchart depicting the study is shown in Fig. 1.

**Table 1:** Baseline characteristics of 259 soft tissue sarcoma patients

	Total set (n = 259)	Training set (n = 183)	Validation set (n = 76)
Age, years	60.71 ± 14.59	60.51 ± 14.61	61.20 ± 14.64
Race			
White	226	160	66
Other	24	14	10
Unknown	9	9	0
Sex			
Male	118	78	40
Female	141	105	36
Tumor site			
Extremity	85	61	24
Other	174	122	52
Margin status			
R0	154	107	47
R1/2	78	57	21
Unknown	27	19	8
Metastasis			
No	120	87	33
Yes	56	35	21
Unknown	83	61	22
Radiotherapy			
No	140	101	39
Yes	73	49	24
Unknown	46	33	13
Histological type			
Leiomyosarcoma	104	72	32
Dedifferentiated liposarcoma	58	43	15
UPS	51	34	17
Myxofibrosarcoma	25	17	8
Synovial sarcoma	10	7	3
Other	11	10	1

*UPS* Undifferentiated pleomorphic sarcoma

### Profiling differentially expressed IRGs (DEIRGs)

To identify tumor-related genes, differential analyses were performed between STS samples from TCGA-SARC and normal muscle and adipose tissues from GTEx in the R software with the “limma” package. The expression differences were assessed according to their log2-fold change (log2 FC) and false discovery rate (FDR). Only genes with |log2FC| > 1 and FDR  < 0.05 were identified as differentially expressed genes (DEGs). According to the IRG dataset from the ImmPort database, DEIRGs were determined by matching 2498 IRGs to the DEGs.

To explore the potential function of the identified DEIRGs, Gene Ontology (GO) functional annotation and Kyoto Encyclopedia of Genes and Genomes (KEGG) pathway enrichment analyses were performed in the R software with the “clusterprofiler” package [24]. The results with an adjusted *p* value < 0.05 were considered as statistically significant.

### Comprehensive analyses of IRG-based clusters in STS patients

Based on the identified DERIGs, unsupervised classification of the 259 STS patients was performed using hierarchical consensus in R. The “ConsensusClusterPlus” package was used to obtain an unbiased and unsupervised outcome [25]. In this study, to ensure high stringency, the optimal number of clusters was determined according to the elbow method and the gap statistic. Additionally, the expression profiles of five common immune checkpoints were extracted from the RNA sequence data of the STS samples. Furthermore, by performing ESTIMATE and CIBERSORT in R, the TME scores and the fraction of 22 types of immune cells were determined. The differences in prognosis, immune checkpoints, TME score, and immune cells were assessed using the Wilcoxon rank-sum test or the Kruskal–Wallis test.

### Identification of overall survival (OS)- and progression free survival (PFS)-related DEIRGs and establishment of two prognostic signatures

To comprehensively understand the prognostic value of DEIRGs, the 259 STS patients were randomly divided into a training set and validation set at a ratio of 7:3 in R. Except for the external validation and subgroup of signatures, all subsequent analyses were performed in the training set. In addition, to better understand the prognostic value of DEIRGs in STS patients, both OS and PFS were analyzed. The OS was defined as the time interval between the day of the first diagnosis and the day of death for any reason, while PFS was defined as the time interval between the day of the first diagnosis and the day of tumor progression or death. All the procedures used for the survival analyses, signature establishment, and signature assessment were performed in R. First, a univariate Cox analysis was performed to determine OS- and PFS-related DEIRGs, which were further selected for the least absolute shrinkage and selection operator (LASSO) analyses using the “glmnet” package [26]. Next, we performed multivariate Cox regression analyses to select candidate OS- and PFS-related DEIRGs to construct two prognostic signatures, termed the OS signature and the PFS signature, respectively. The coefficients of all DEIRGs in the final signatures were confirmed simultaneously and were used to calculate risk scores for each STS patient in the training set. The risk score was calculated as follows:

$$ Risk{\text{ Score = }}\sum\limits_{i = 0}^{n} {\beta_{i} {\text{ * G}}_{i} } $$

$\beta_{i}$ is the coefficient of gene $i$ in the multivariate Cox analysis; ${\text{G}}_{i}$ is the expression value of gene $i$; and $n$ is the number of genes in the signature.

To assess the performance of our signatures, the “survivalROC” package was used to generate receiver operating characteristic (ROC) curves at 1, 2, and 3 years, and the corresponding time-dependent area under the curves (AUCs) were calculated simultaneously. The best cutoff value of the risk score was then identified by X-tile software, and all patients were divided into a high- or low-risk group [27]. Kaplan–Meier (K-M) survival curves with the log-rank test were generated to find differences in the OS and PFS between the two groups.

### Validation of the prognostic signatures

External validation is critical when establishing prognostic signatures. To validate the two prognostic signatures, the expression profile data of the genes included in the signatures were extracted from the validation set and substituted into the equations for risk score calculation. According to the same cutoff value identified in the training set, all patients in the validation set were divided into the high- or low-risk group. The prediction accuracy of the signatures was verified by ROC and K-M survival curves.

### Subgroup analyses of signatures

To confirm that a DEIRG-based signature can perform consistently in several subgroups, subgroup analyses were performed regarding age, sex, tumor site, and margin status group; these analyses were performed in both the training and validation sets. First, according to the clinical data, all patients were divided into subgroups. The K-M curves in several subgroups were then generated to reveal differences in prognosis between the two risk groups.

### Development of nomograms integrating IRG signatures and clinical variables

Clinical variables, including age, race, sex, tumor site, margin status, metastatic status, and radiotherapy were obtained from the cBioPortal database [22]. Univariate Cox analysis combining the signature and clinical variables was performed for STS patients in the training set, and factors with a P < 0.05 were incorporated into the multivariate Cox analyses to select the independent prognostic variables. Next, two prognostic nomograms were established by the “rms” package in R based on the independent prognostic factors for predicting OS and PFS in STS patients. The concordance index (C-index) was used to assess the discrimination of the two nomograms, and calibration curves were generated to evaluate the concordance between actual and nomogram-predicted outcomes.

### Identification and validation of TF-IRG regulatory network

The transcription factor (TF) set was downloaded from Cistrome Cancer (http://cistrome.org/CistromeCancer/). According to the DEGs identified in the differential analyses and the TF set downloaded from the Cistrome Cancer, differently expressed TFs (DETFs) were extracted. GO and KEGG enrichment analyses were performed in R to identify the potential functions and related pathways of the DETFs [24]. Univariate Cox analysis was then used to determine OS- and PFS-related DETFs. To explore the potential TF-IRG regulatory network in STS patients, the correlation between OS-related DETFs and OS-related DEIRGs was measured by Pearson correlation analysis. The correlation between PFS-related DETFs and PFS-related DEIRGs was processed by the same method. Correlation analyses results with P < 0.05 and |r| > 0.2 were considered as statistically significant [28]. Two TF-IRG regulatory networks were illustrated using Cytoscape (3.7.2) [29].

### Statistical analyses

All statistical analyses were performed in R (version 3.6.1). Unpaired Student’s t-test, the Wilcoxon rank-sum test, ANOVA, and the Kruskal–Wallis test were used for the comparison of continuous variables. The Chi square test and Fisher’s exact test were used to compare categorical variables. Pearson analysis was used for the correlation analyses. A P-value  < 0.05 (two-tailed) was considered to indicate statistical significance.

## Results

### Patient characteristics

**Fig. 2:**
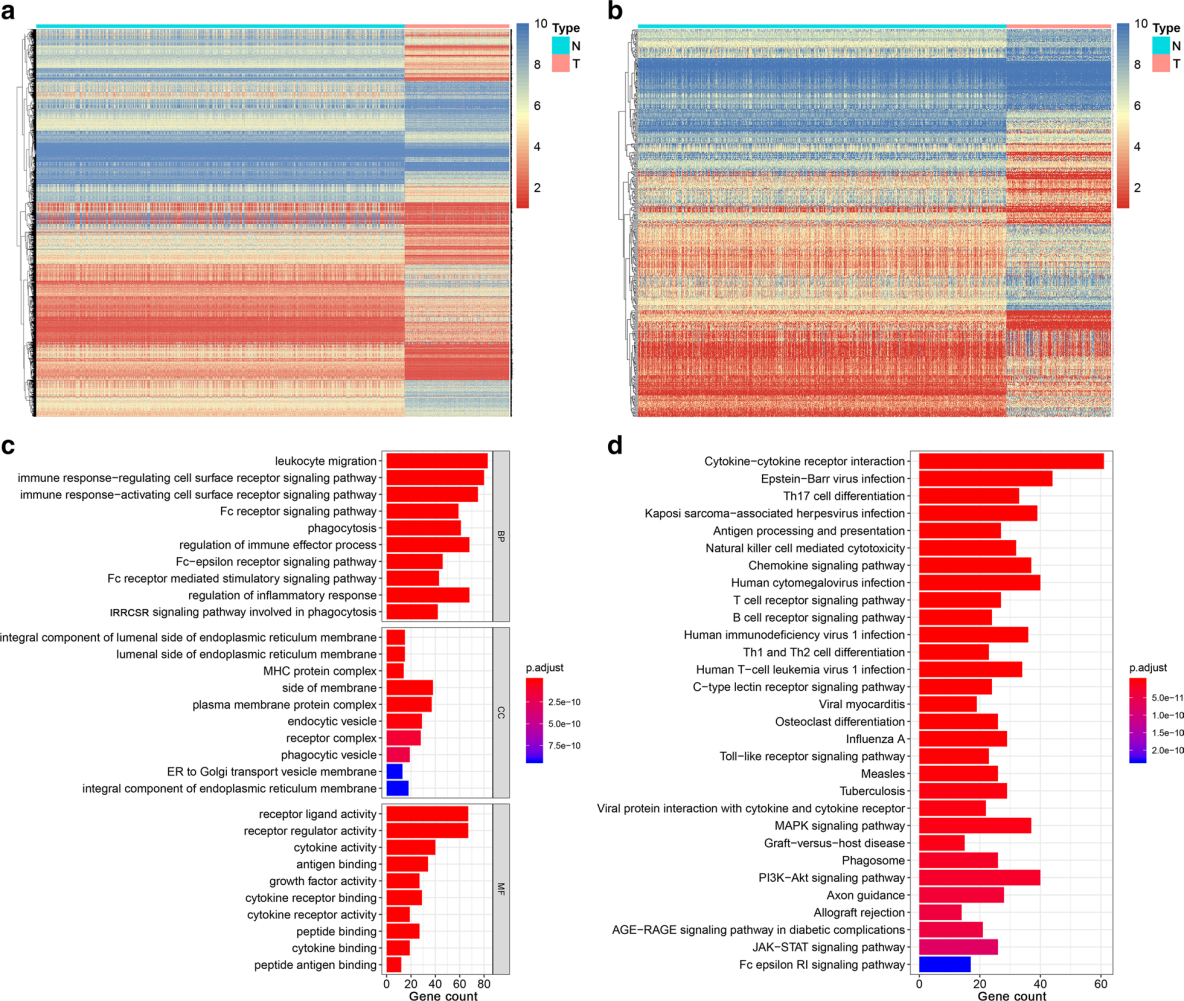
Expression and enrichment analyses of DEGs in sarcoma patients. **a** Heat map of 5610 DEGs; **b** Heat map of 364 DEIRGs; **c** Bar chart showing the top ten most significant terms in the GO analyses, including BP, CC, and MF; the x-axis refers to the number of genes that are enriched in the corresponding function. **d** Bar chart showing the top 30 most significant terms in KEGG pathway enrichments of DEIRGs; the x-axis refers to the number of gene that are enriched in the corresponding function. DEGs: Differentially expressed genes; DEIRGs: Differentially expressed immune-related genes; *GO* Gene ontology, *BP* Biological process, *CC* Cellular component, *MF* Molecular function, *KEGG* Kyoto Encyclopedia of Genes and Genomes

In total, 259 STS tumor samples and 911 normal muscle and adipose samples were included and their expression profiles were examined. After screening, a total of 5610 DEGs were identified (Fig. 2a). Next, 364 DEIRGs were selected by matching IRGs to DEGs, including 232 upregulated and 132 downregulated genes (Fig. 2b). To gain a better understanding of how these DEIRGs may drive STS development, GO and KEGG enrichment analyses were performed. Functional annotations revealed that DEIRGs were mainly involved in leukocyte migration, the immune response-regulating cell surface receptor signaling pathway, and the immune response-activating cell surface receptor signaling pathway based on the top three terms identified in the GO analyses (Fig. 2c). KEGG analysis found that DEIRGs were mainly associated with cytokine–cytokine receptor interaction, Epstein-Barr virus infection, and Th17 cell differentiation (Fig. 2d). The enrichment analyses indicated that DEIRGs might not only play a vital role in tumorigenesis but are also important in the tumor immune microenvironment.

### IRG-based clusters significantly associated with immune features

**Fig. 3:**
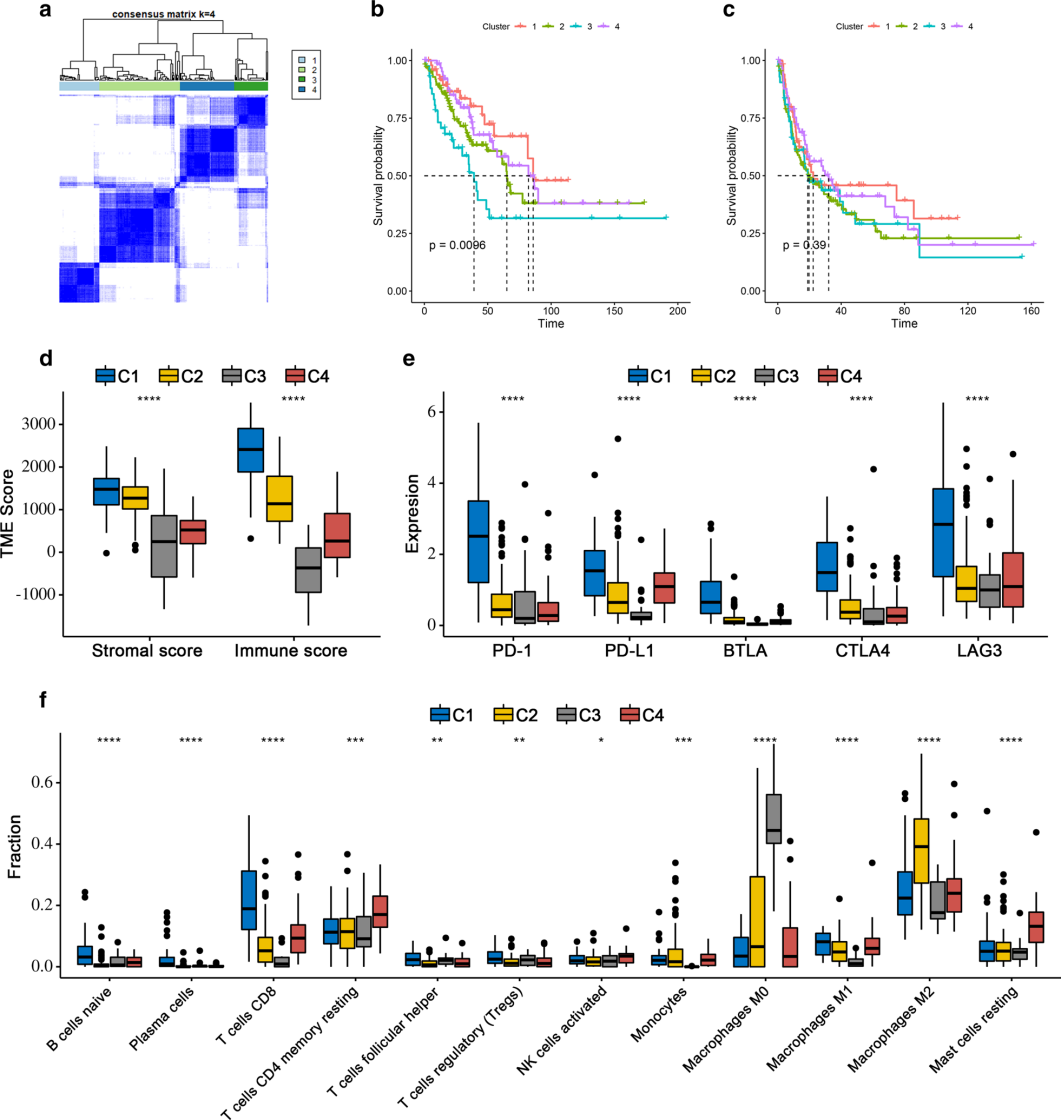
Comprehensive profiling of IRG-based clusters in sarcoma patients. **a** The consensus matrix heatmap defined four clusters from the 259 sarcoma patients. **b** Survival analysis showed that IRG-based clusters were significantly associated with OS. **c** Survival analysis showing that IRG-based clusters were not significantly associated with PFS. **d** Tumor microenvironment scores of the four clusters. **e** The expression levels of five immune checkpoints (PD-1, PD-L1, BTLA4, CTLA4, and LAG3) in the four clusters. **f** Histogram of the infiltration levels of 12 significant immune cells in the four clusters. *P < 0.05; **P < 0.01; ***P < 0.001; ****P < 0.0001. *IRG* Immune-related gene, *OS* Overall survival, *PFS* Progression-free survival

As shown by the DEIRG profiling, DEIRGs were markedly heterogeneous among the STS patients. To gain insight into the molecular heterogeneity of STS and explore whether IRGs presented discernable patterns in STS, we performed unsupervised consensus analysis of all samples based on 364 DEIRGs. Combining the elbow method and the gap statistic method enabled us to determine the optimal number of clusters (K = 4) (Fig. 3a). According to the consensus matrix heatmap, four IRG-based clusters were determined as follows (Fig. 3a): C1 (n = 50, 19.3%), C2 (n = 100, 38.6%), C3 (n = 42, 16.2%), and C4 (n = 67, 25.9%). Survival analyses showed that C3 was associated with worse OS prognosis (Fig. 3b), but there were no significant differences in PFS among the four clusters (Fig. 3c).

Given the potential function of DEIRGs in immune regulation shown in the enrichment analyses, we further investigated whether IRG-based clusters show different immune patterns. The Kruskal–Wallis tests indicated that TME scores, including immune and stromal scores, were significantly associated with IRG clusters (Fig. 3d). In addition, the distribution of immune checkpoints, including PD-1, PD-L1, BTLA-4, CTLA4, and LAG3, were not random (Fig. 3e). For example, samples classified as C3 were more enriched in immune checkpoints and had the lowest immune scores of the four clusters and may be more insensitive to immunotherapy.

To assess the correlation between IRGs and additional immune infiltration characteristics, we compared the levels of 22 types of immune cells among the four clusters. There were significant differences among the four clusters for the following cell types: B cell native, plasma cells, T cells CD8, T cells CD4 memory resting, T cells follicular helper, T cells regulatory (Tregs), NK cells activated, monocytes, Macrophages M0, Macrophages M1, Macrophages M2, and Mast cells resting (Fig. 3f).

### Prognostic value of DEIRGs in STS

**Fig. 4:**
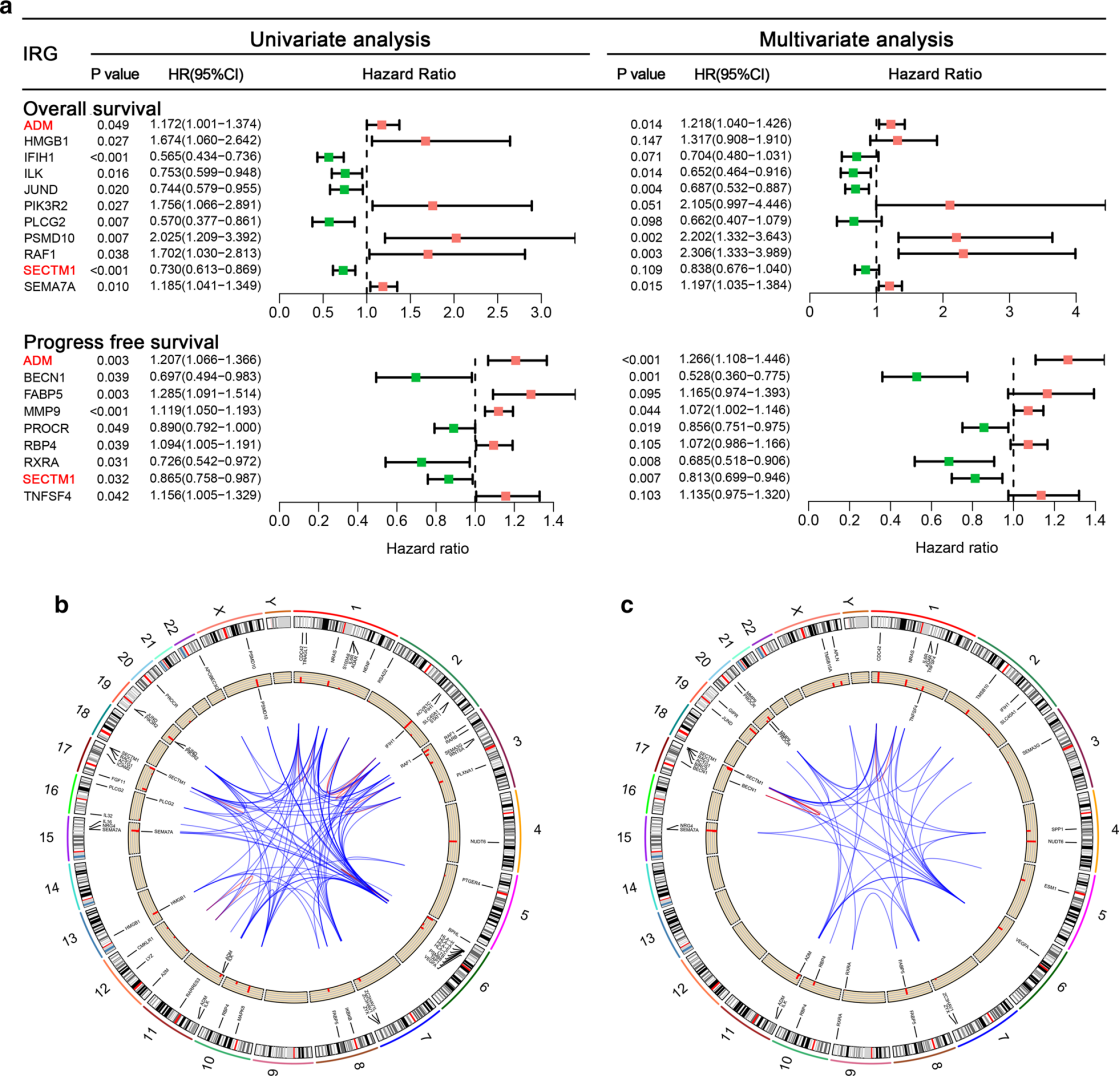
Prognostic analyses for sarcoma patients. **a** Forest plot showing the results of the univariate and multivariate Cox analyses for 18 IRGs that were incorporated into the signatures. Two overlapping genes are highlighted in red. **b** Circos plot showing the location of OS-related IRGs. From outside to inside: 23 chromosomes, significant IRGs in the univariate Cox analysis, HR value of corresponding IRGs in the univariate Cox analysis, IRGs incorporated into the OS signature, and gene–gene interactions. **c** Circos plot showing the location of PFS-related IRGs. From outside to inside: 23 chromosomes, significant IRGs in the univariate Cox analysis, HR value of corresponding IRGs in the univariate Cox analysis, IRGs incorporated into the PFS signature, and gene–gene interactions. *IRGs* Immune-related genes, *OS* Overall survival, *PFS* Progression-free survival

The detection of robust markers for the early diagnosis of tumors and potential therapeutic targets remains a crucial issue for clinical practice. Recent findings have shown that the aberrant expression of IRGs occurs in early-stage tumors and can serve as prognostic biomarkers in several cancers. Hence, we further studied the potential prognostic value of DEIRGs in STS patients. Univariate Cox regression analysis identified 57 and 33 DEIRGs to be significantly associated with OS and PFS, respectively (Additional file 2 and Additional file 3). In the subsequent LASSO regression analysis, 20 and 16 DEIRGs were identified as OS- and PFS-related factors, respectively (Additional file 4: Figure S1 and Additional file 5: Figure S2). Next, multivariate Cox analysis was performed, and 18 DEIRGs were used to construct two prognostic signatures, including nine DEIRGs for the OS signature only, seven DEIRGs for the PFS signature only, and two overlapping DEIRGs (Fig. 4a). Two circos plots were generated to show the location of prognostic genes in the chromosome (Fig. 4b, c). The formula for the OS signature was as follows: Risk score = −0.37552*JUND + 0.27513*HMGB1 + 0.74431*PIK3R2 + 0.78955*PSMD10 −0.42760*ILK + 0.19689* ADM −0.17645*SECTM1 + 0.17991*SEMA7A−0.41175*PLCG2 + 0.83549* RAF1−0.35149*IFIH1. The PFS signature was as follows: Risk score = −0.15562* PROCR + 0.15235* FABP5 + 0.06963* RBP4 −0.37785* RXRA + 0.23565* ADM −0.20687* SECTM1 + 0.12620* TNFSF4 −0.63813* BECN1 + 0.06908* MMP9.

**Fig. 5:**
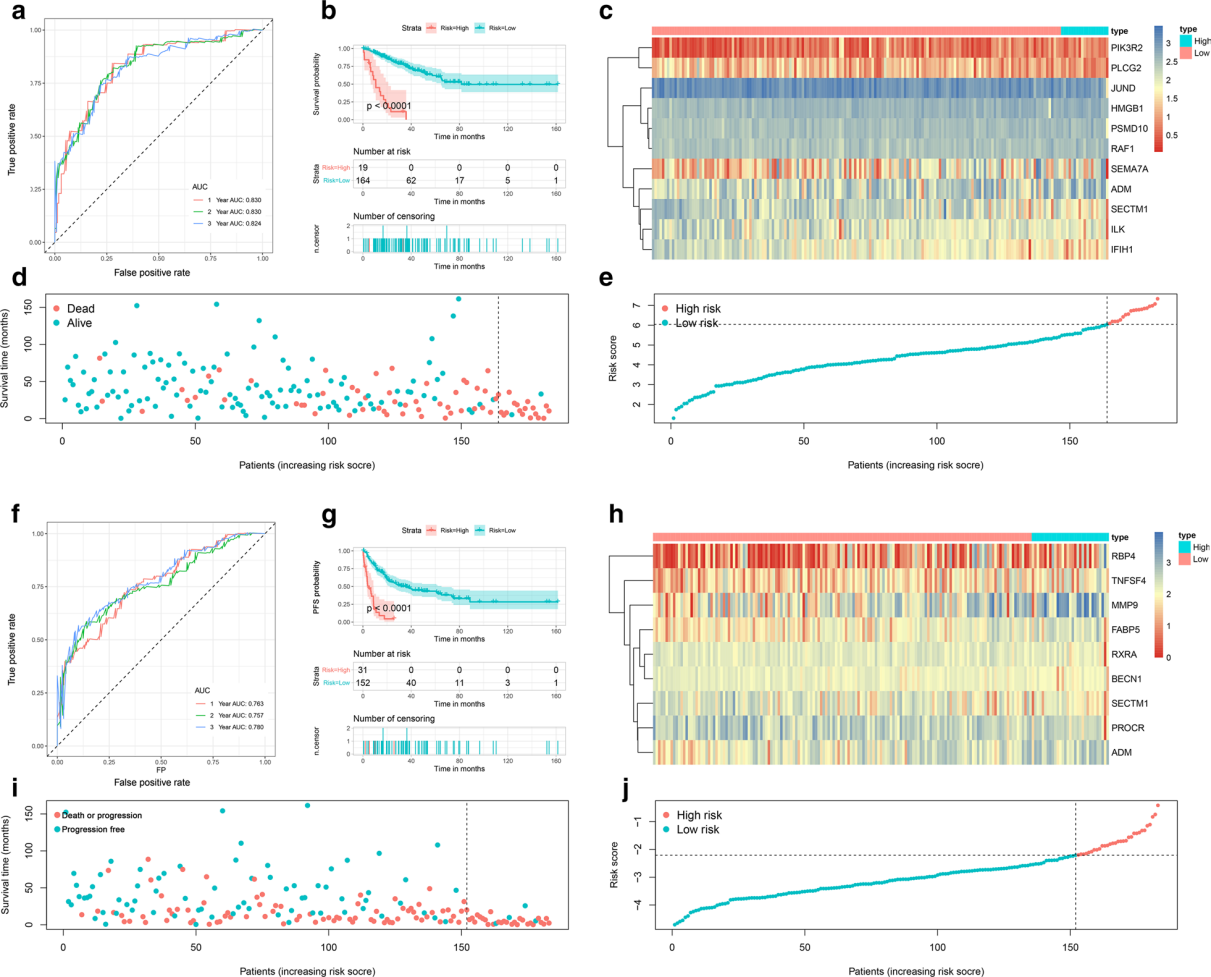
Establishment of an 11-DEIRG-based OS signature and a 9-DEIRG-based PFS signature. (**a**, **f**) Time-dependent ROC curves of the OS and PFS signatures at 1, 2, and 3 years. (**b**, **g**) Kaplan–Meier survival curves showing the differences in OS and PFS between low-risk and high-risk patients. (**c**, **h**) Differential gene expression between the high-risk and low-risk groups in the OS and PFS signatures. (**d**, **i**) OS and PFS scatter plots for sarcoma patients. (**e**, **j**) Risk score distribution of patients with the OS and PFS signatures. DEIRGs: Differentially expressed immune-related genes; *OS* Overall survival, *PFS* Progression-free survival, *ROC* Receiver operating characteristic

The time-dependent ROC curves showed that the AUCs of the OS signature for predicting 1, 2, and 3 year OS were 0.830, 0.830, and 0.824, respectively (Fig. 5a). Subsequently, according to the optimal risk score cutoff identified by X-tile, STS patients were stratified into high-risk (n = 19) and low-risk groups (n = 164). The K-M curves indicated that patients in the high-risk group had a worse OS than those in the low-risk group (Fig. 5b). Similarly, the time-dependent ROC curves of the PFS signature were also generated. The AUCs of the signature for predicting 1, 2, and 3 year PFS were 0.763, 0.757, and 0.780, respectively (Fig. 5f). The optimal risk score cutoff was also identified by X-tile, and 31 and 152 patients were stratified into high-risk and low-risk groups, respectively. The patients with lower risk scores had favorable PFS (Fig. 5g). These results revealed that both signatures were valuable tools for predicting the prognosis of STS patients. Moreover, to more clearly visualize differences in the prognosis and gene expression patterns, heatmaps (Fig. 5c, h), survival status plots (Fig. 5d, j), and risk score plots (Fig. 5e, j) were generated.

### External validation of the prognostic signatures

**Fig. 6:**
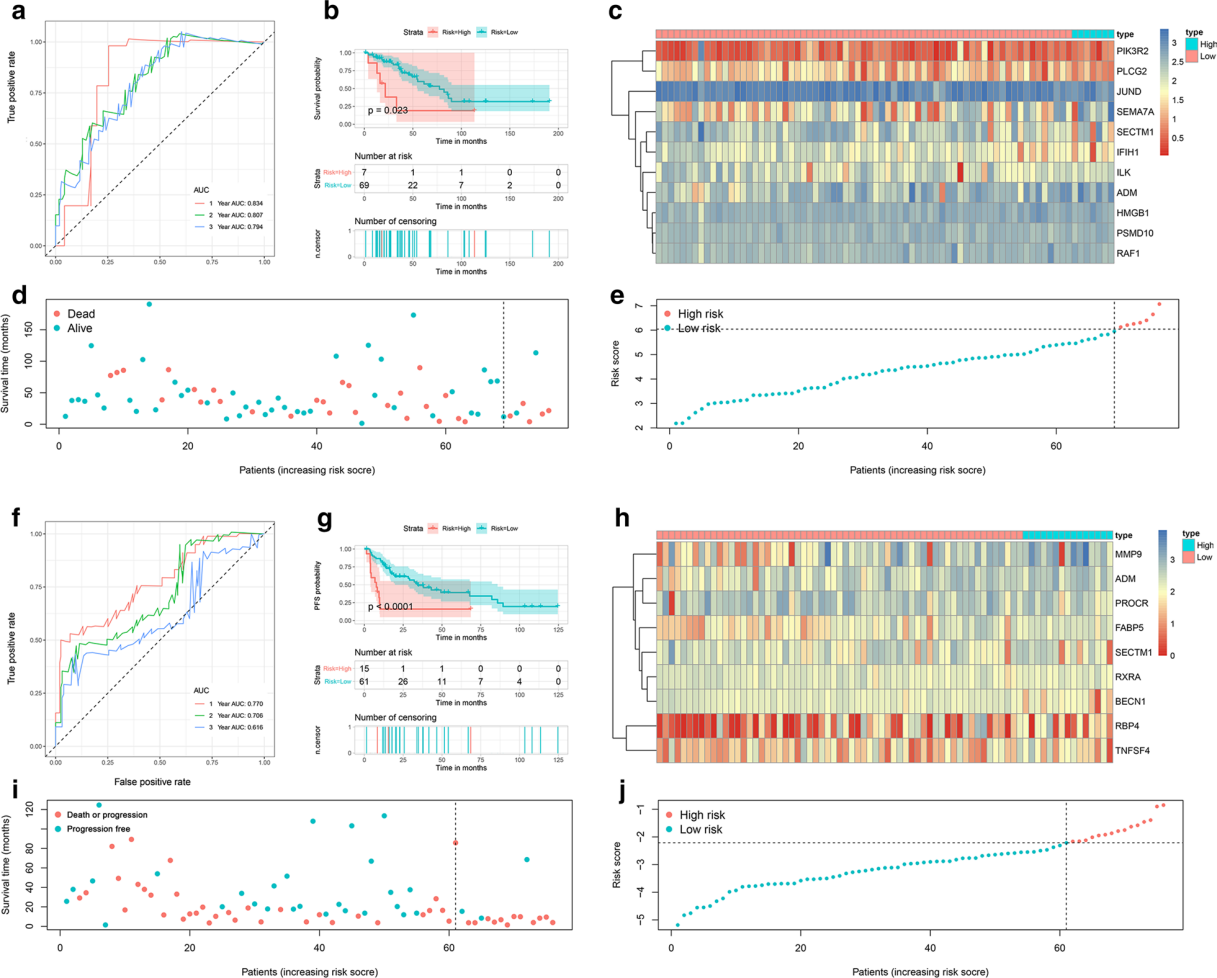
External validation of OS and PFS signatures. (**a, f**) Time-dependent ROC curves of the OS and PFS signatures at 1, 2, and 3 years. (**b, g**) Kaplan–Meier survival curves showing the differences of OS and PFS between low-risk and high-risk patients. (**c, h**) Gene expression levels of the high-risk and low-risk groups with the OS and PFS signatures. (**d, i**) OS and PFS scatter plots for sarcoma patients. (**e, j**) Risk score distribution of patients with the OS and PFS signatures. *OS* Overall survival, *PFS* Progression-free survival, *ROC* Receiver operating characteristic

External validation is crucial for validating the applicability of a prognostic signature. A total of 76 independent STS patients were included in this validation. First, according to the formulas generated in the training set, the risk scores were calculated, including the risk score based on the OS signature and the risk score based on the PFS signature. The ROC curves revealed that the discrimination of both signatures in the validation set were favorable, with the AUC ranging from 0.616-0.834 (Fig. 6a, f). The optimal risk score cutoff identified in the training set was used to stratify the patients into high-risk and low-risk groups. The K-M survival curves showed significant differences in both OS and PFS between the two groups, which was consistent with the training set (Fig. 6b, g). Additionally, two heatmaps, the survival status distribution, and the risk score distribution were also generated for the validation set to illustrate the differences between the high-risk and low-risk groups (Fig. 6c–e and h–j). To more comprehensively evaluate our signatures, ROC curves, survival curves, heatmaps, the survival status distribution, and the risk score distribution were also generated for the total TCGA cohort. Both signatures showed satisfactory performance in the total cohort (Additional file 5: Figure S2).

### Subgroup analyses of prognostic signatures

To investigate the applicable STS population of the DEIRG-based signatures, survival analyses with log-rank tests were further performed in subgroups based on several clinical variables in both the training and validation sets (Additional file 6: Figure S3). We validated the stability of our signatures by age (< 60 or ≥ 60), sex (male or female), tumor site (extremity or other), and margin status (R0 or R1-2) (Additional file 6: Figure S3). K-M survival analyses indicated that both the OS and PFS signatures were robust prognostic models for all subgroups, indicating that our prognostic signatures that were generated based on DEIRGs have strong robustness to predict prognosis in different patient subgroups (all p < 0.001).

### Establishment of nomograms based on DEIRG signature and clinical parameters

**Fig. 7:**
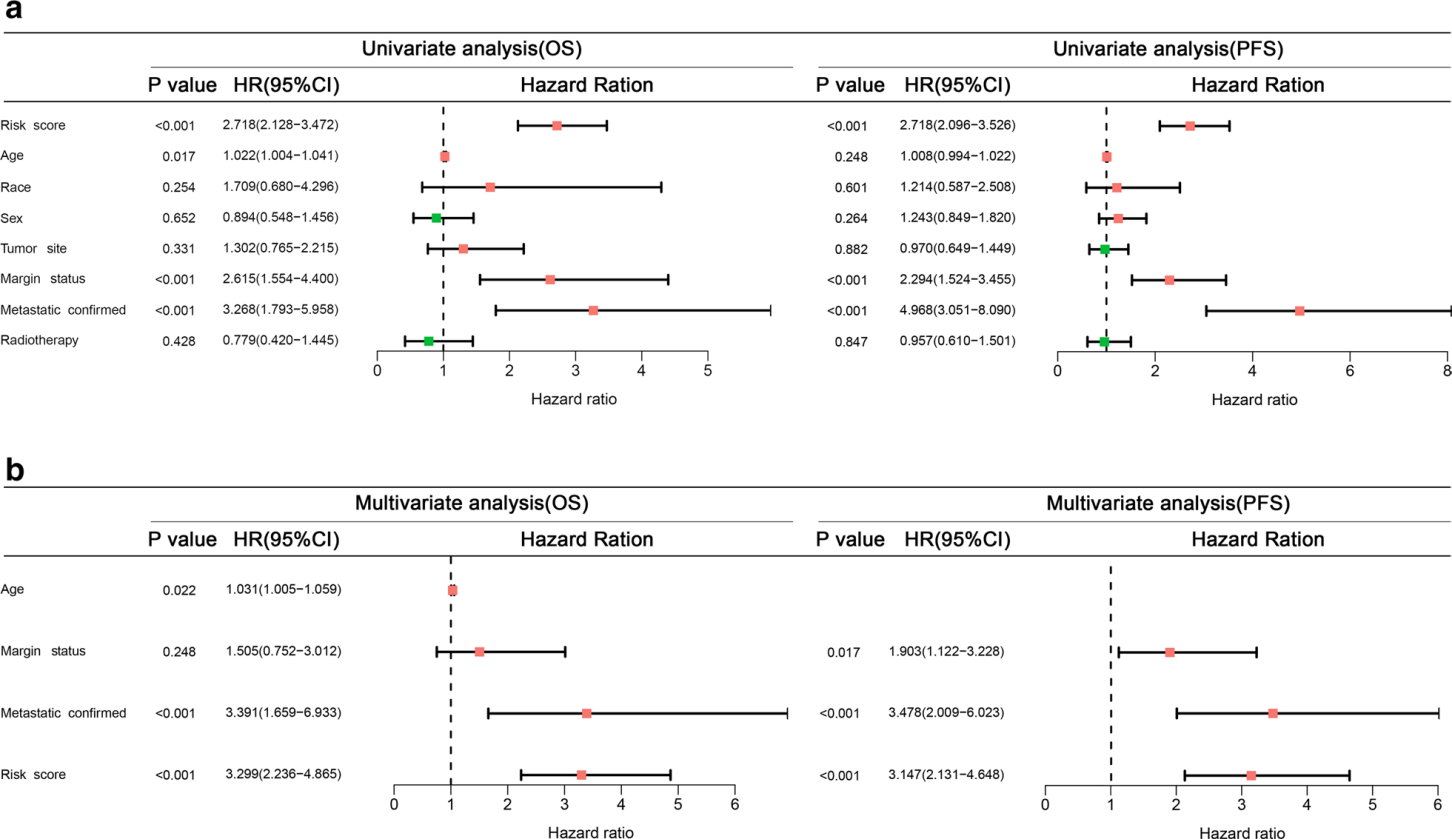
Survival analyses showed that both OS and PFS signatures are independent factors to predict the prognosis of sarcoma patients. **a** Univariate Cox analyses of the DEIRG-based prognostic signature and clinical variables. **b** Univariate Cox analyses of the significant variables in the univariate Cox analyses. *DEIRG* Differentially expressed immune-related genes, *OS* Overall survival, *PFS* Progression-free survival

Two comprehensive models combining independent clinical parameters were constructed to improve the clinical application of our prognostic signatures. First, we performed univariate and multivariate Cox analyses to assess independent OS and PFS prognostic variables (Fig. 7a,b). Four independent OS-related variables were identified, including risk score, age, margin status, and metastatic status (Fig. 7b). The risk score, margin status, and metastatic status were identified as independent PFS-related variables (Fig. 7b). These results demonstrated that both DEIRG-based signatures can be used independently to predict the prognosis of STS patients.

**Fig. 8:**
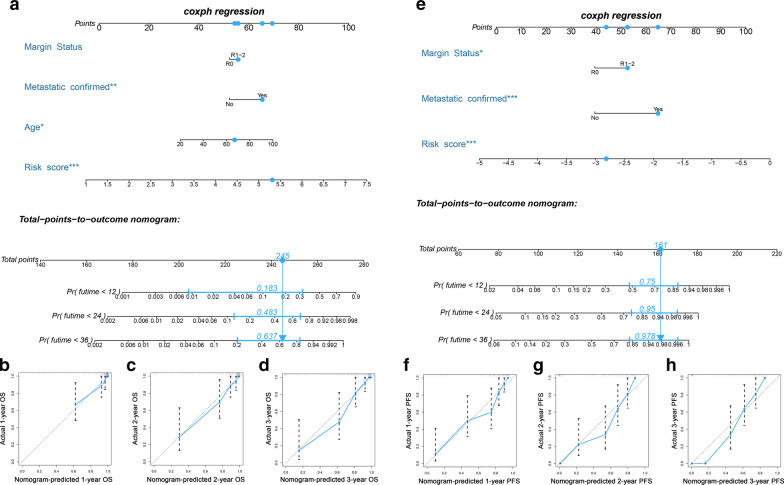
Development of two nomograms combining DEIRG-based signature and independent prognostic clinical variables to predict OS and PFS in sarcoma patients. **a** Nomogram of OS combining the OS signature and three clinical variables of sarcoma patients. (**b-d**) Calibration curves of the nomogram at 1, 2, and 3 years. (**e**) Nomogram of PFS combining the PFS signature and two clinical variables of sarcoma patients. In the two nomograms, a patient example marked in sky-blue is shown. (**f–h**) Calibration curves of the nomogram at 1, 2, and 3 years. *DEIRGs* Differentially expressed immune-related genes, *OS* Overall survival, *PFS* Progression-free survival

Subsequently, based on the independent prognostic variables, two novel nomograms were established to predict OS and PFS (Fig. 8a, e). The C-indexes were 0.879 (95%CI 0.832–0.926) and 0.825 (95%CI 0.776–0.874) for the OS and PFS nomogram, respectively. The favorable calibration of the nomograms indicated that the nomogram-predicted outcomes were highly consistent with the actual observations (Fig. 8b–d and f–h).

### Construction of TF-IRG regulatory networks

**Fig. 9:**
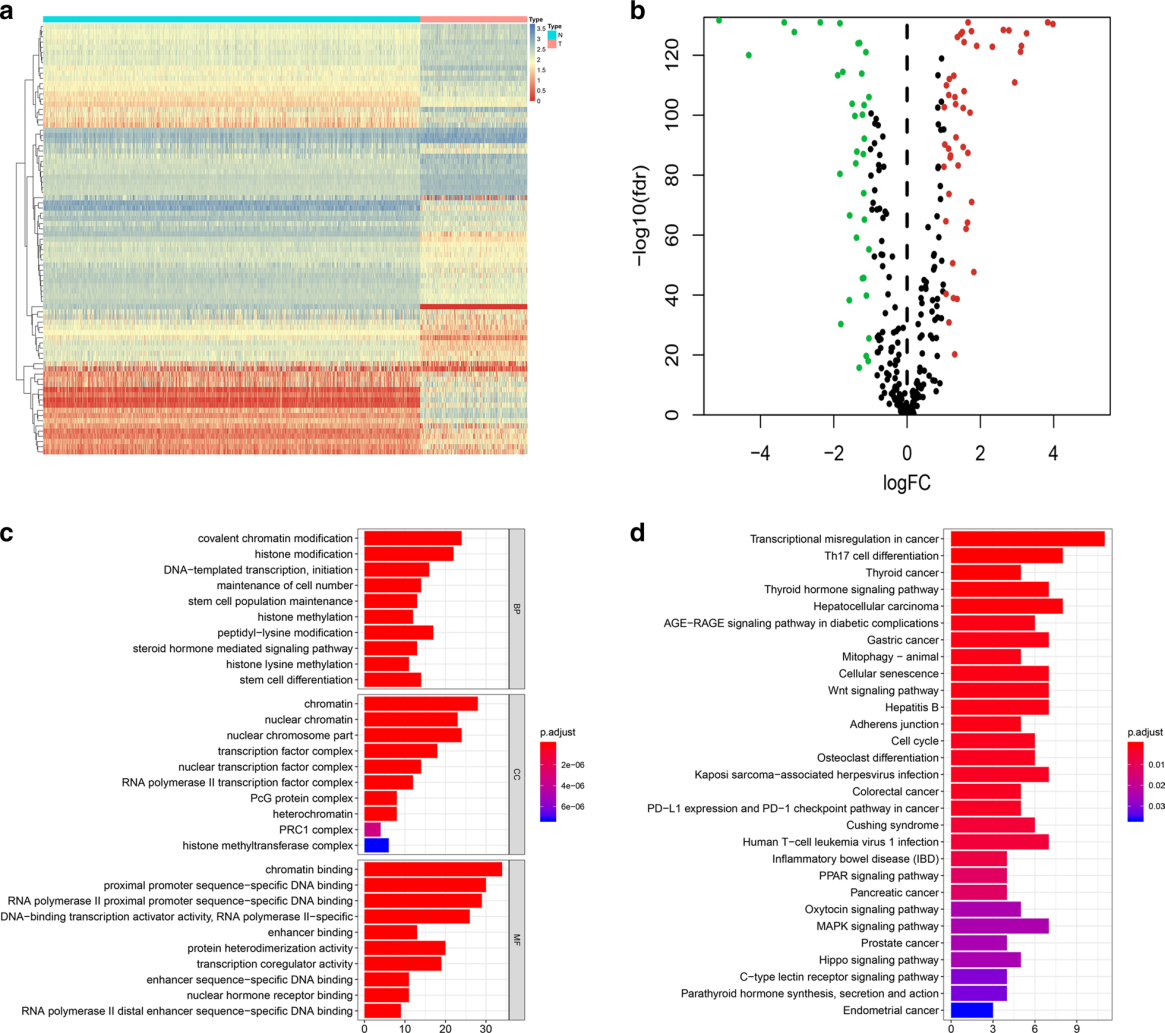
Differential analysis and functional annotations of DETFs. **a** Heatmap showing the differential expression of 83 DETFs between sarcoma and normal tissues. **b** Volcano plots of 83 DETFs; the red plot shows upregulated genes and the green plot shows downregulated genes. **c** Bar chart of top ten most significant terms in BP, CC, and MP for 83 DETFs. **d** Bar chart of the top 30 most significant terms in the KEGG pathway enrichment for DETFs. DETFs: Differentially expressed transcription factors; *BP* Biological process, *CC* Cellular component, *MF* Molecular function, *KEGG* Kyoto encyclopedia of genes and genomes

**Fig. 10:**
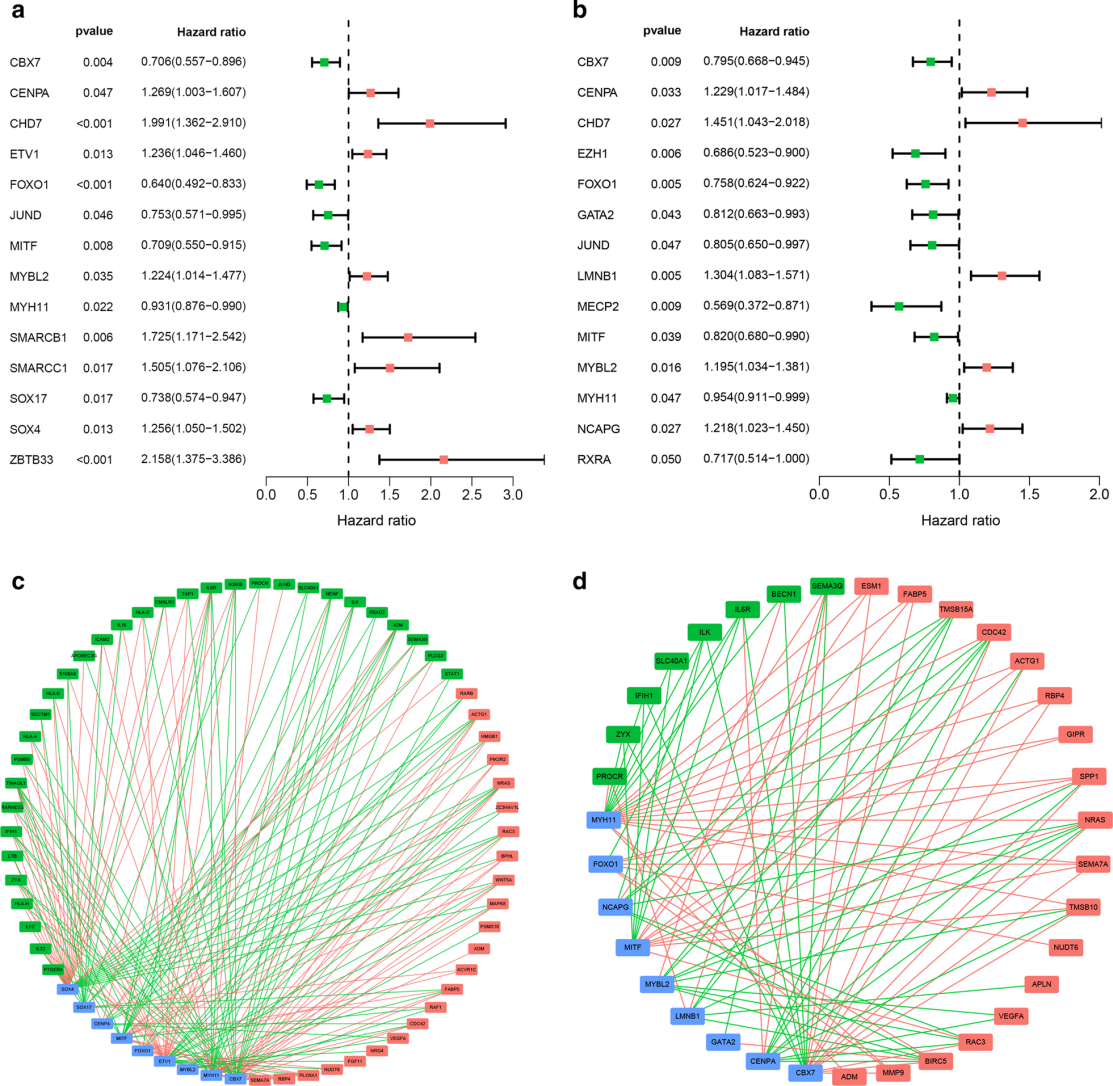
Two TF-IRG regulatory networks. **a** Fourteen DETFs were determined to be OS-related DETFs; **b** Fourteen DETFs were determined to be PFS-related DETFs. **c** The regulatory network of OS-related DETFs and OS-related DEIRGs. **d** The regulatory network of PFS-related DETFs and PFS-related DEIRGs. The blue rectangle represents OS-related DETFs, the green rectangle represents favorable DEIRGs, and the red rectangle represents worse DEIRGs. *TF* Transcription factor, *IRG* Immune-related gene, *DETFs* Differentially expressed transcription factors, *OS* Overall survival, *PFS* Progression-free survival

Differential analysis confirmed 83 DETFs between the STS samples and normal tissue samples (Fig. 9a, b). GO analysis functional annotation revealed that DETFs were mainly involved in covalent chromatin modification, chromatin, and chromatin binding in BP, CC, and MF, respectively (Fig. 9c). KEGG pathway analysis showed that DETFs were mainly enriched in transcriptional misregulation in cancer, Th17 cell differentiation, and hepatocellular carcinoma (Fig. 9d). Univariate Cox proportional hazard modeling was used to identify prognosis-related DETFs, and 14 DETFs were determined as OS- and PFS-related DETFs, respectively (Fig. 10a, b). The correlation between the expression levels of 14 OS-related DEIRGs and 57 OS-related DEIRGs and between the expression levels of 14 PFS-related DEIRGs and 33 PFS-related DEIRGs were analyzed by Pearson correlation analysis. Two TF-IRG regulatory networks were constructed and visualized (Fig. 10c, d).

**Fig. 11:**
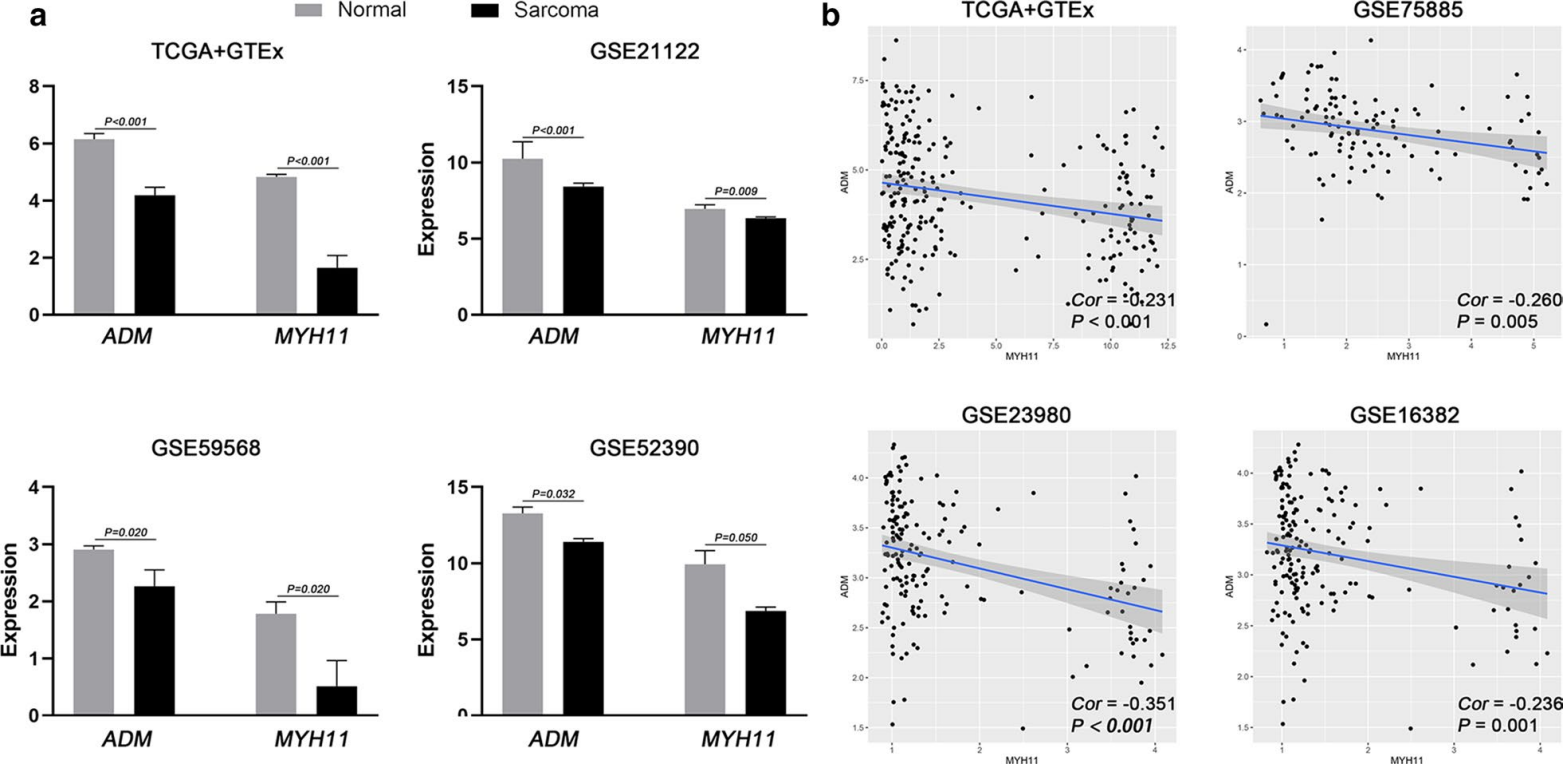
Validation of the crucial MYH11-ADM regulatory network. **a** Four datasets, including one dataset from GTEx and TCGA and three independent datasets from the GEO database, showed that both MYH11 and ADM were significantly lower in sarcoma tissue. **b** Four datasets, including one dataset from GTEx and TCGA and three independent datasets from the GEO database, showed a negative association between MYH11 and ADM in sarcoma samples. *GTEx* Genotype-Tissue Expression, *TCGA* The Cancer Genome Atlas, *GEO* Gene Expression Omnibus

Interestingly, in the TF-IRG regulatory network analysis, a crucial regulatory network was identified—MYH11-ADM. As a DEIRG, ADM was determined to be an independent OS- and PFS-related gene in the multivariate Cox analysis and was therefore incorporated into both signatures (Fig. 4a). In addition, as a DETF, MYH11 showed significant associations with both OS and PFS (Fig. 10). Therefore, we further studied the expression patterns of MYH11 and ADM and the correlation between MYH11 and ADM. Several gene expression profiles of STS and adjacent normal samples were retrieved from several datasets. Compared with normal tissue, the expression levels of MYH11 and ADM were significantly lower in the STS samples (Fig. 11a), which was consistent with our above findings. In addition, the regulatory pattern of MYH11 and ADM was also successfully verified in three independent datasets (Fig. 11b). ADM and MYH11 were identified as abnormally expressed genes and are considered to be prognostic biomarkers for STS patients. A regulatory relationship may exist between them.

## Discussion

STSs are a rare group of highly heterogeneous cancers with a high rate of metastasis of up to 40–50% [8]. More than 50 different histological types with distinct clinical outcomes and biological behaviors complicate the prognostic prediction for STS patients [1, 2]. Hence, the present challenge was to identify precise biomarkers for prognosis assessment and targeted therapy in STS patients. In this investigation, we revealed insights into the role of IRGs in STS patients. A total of 364 DEIRGs were identified as candidate prognostic biomarkers, and functional annotations identified the potential mechanisms of these DEIRGs. Additionally, DEIRG-based clusters identified by unsupervised consensus analysis revealed that DEIRGs presented discernable patterns in STS and had significant associations with immune features. Importantly, we established an OS-prognostic signature based on 11 key DEIRGs and a PFS-prognostic signature based on nine DEIRGs. Both of these DEIRG-based signatures were successfully validated in an independent validation set. The robustness of these two models was supported by the significant associations found between the risk score, levels of immune cell infiltration, and the expression levels of immune checkpoints. In addition, two comprehensive nomograms incorporating the DEIRG-based prognostic model and clinical parameters were constructed to improve the clinical application. By entering the score of each parameter, these nomograms may enable clinicians to estimate the OS and PFS for each STS patient. Finally, 14 OS-related DETFs and 14 PFS-related DETFs were selected, and two TF-IRG regulatory networks were generated to illustrate the relationship between prognostic TFs and IRGs.

Remarkable advances in our understanding of the tumor microenvironment and the immune system have resulted in significant breakthroughs in cancer immunotherapy [30, 31]. Novel immune infiltrate-based classification of sarcoma identified by integrating immune cell populations and tumor cell characteristics has shown promising prognostic ability [32]. IRG expression is connected to the immune infiltration level, key gene mutations, and chemosensitivity [33–35]. IRGs have been identified as effective prognostic biomarkers in ovarian cancer [36], non-squamous non-small cell lung cancer [37], and renal papillary cell carcinoma [38]. To the best of our knowledge, this is the first study to combine the entire set of IRGs with STS data from the perspective of OS and PFS. Our findings may greatly improve the precise classification and individual treatments of STS patients.

We first selected 364 DEIRGs and 83 DETFs from 259 STS patients and 911 normal tissue samples. Enrichment analysis revealed that the DEIRGs were primarily involved in leukocyte migration, the immune response-regulating cell surface receptor signaling pathway, and the immune response-activating cell surface receptor signaling pathway, the dysregulation of which are key factors in tumor initiation and development [39, 40]. Cell surface receptors have long been considered to be significant at all stages of tumorigenesis, and the combined participation of integrins and MMPs is required for the invasion of tumor cells into surrounding tissues and metastasis [39, 41]. The KEGG pathway analysis provided additional evidence that the associations between DEIRGs may have clinical application potential in cancer. It has been estimated that Epstein-Barr virus infection is associated with approximately 200,000 malignancies each year [42], and EBS appears to dysregulate the expression of TCL1-family genes, leading to several typical lymphocyte cancers [43]. Th17 cells are a subset of CD4 + T cells, and high levels of tumor-infiltrating Th17 cells are correlated with lymph node metastases and have a negative impact on the postoperative survival of cancer patients [44].

To be the best of our knowledge, this is the first study to perform a systematic analysis of IRG-based clustering of STS. Four clusters were identified in our research. The TME score, immune checkpoints, and immune cells were confirmed to be unevenly distributed among the four clusters. As the worst-prognosis cluster, C3 had the lowest immune score, lowest stromal score, and several immune checkpoints. Although immunotherapy has been widely studied in lung cancer [45], gastrointestinal cancer [46, 47], melanoma [48, 49], and renal cancer [50], its application in STS has received little research attention. This might be attributed to the unclear role of immune checkpoints in STS patients. Although our research indicated that the cluster with the lowest PD-L1 expression level showed a worse prognosis, and the same conclusion was observed at the protein level [51], some studies have come to the opposite conclusion [52, 53]. For CTLA4, LAG3, and BTLA, some level of correlation between the expression level and cluster was also observed. For precision medicine, a further study based on a larger cohort with better controls should be performed to clearly elucidate the role of immune checkpoints in STS patients.

In this study, the prognostic value of DEIRGs was also investigated. An OS-prognostic signature based on 11 DEIRGs and a PFS-prognostic signature based on nine DEIRGs were constructed and then successfully validated in an independent set. The differences in OS and PFS status between patients with low and high scores were notable in the two sets. The time-dependent AUCs indicated that the models performed well in predicting the prognosis of STS patients. Among the DEIRGs included in the two prognostic models, ADM and SECTM1 were found to be associated with both OS and PFS, which may have great clinical significance. ADM, a vasodilating peptide known as a regulator in the pathophysiology of cardiovascular disease, was recently found to have the ability to promote the growth of subcutaneously transplanted sarcoma 180 tumor cells, and ADM inhibitors were shown to be useful for the management of sarcoma growth [54]. In addition, as a receptor regulatory protein of AMD, the overexpression of RMAMP2 suppressed the adhesion of sarcoma cells to endothelial cells and metastasis via vascular integrity [55]. Interestingly, the MYH11-ADM regulatory network has an important role in STS patients. Both proteins were significantly associated with OS and PFS in STS patients, and there is likely an important connection between them. Although details of this relationship within the tumor are unclear, our study lays the foundation for future research directions. Importantly, although both OS and PFS were studied, PFS is not a good surrogate for OS for patients who receive immunotherapy [56]. Given the rapid development of therapeutics in oncologic research, the assessment of both outcomes (OS and PFS) is essential for clinical and policy decision-making.

Regarding the other overlapping prognostic DEIRG, SECTM1 is often referred to as a ligand of CD7 and has rarely been studied. Recently, Wang et al. [57] showed that SECTM1 produced by tumor cells could bind to CD7 and significantly promote monocyte migration by activating the PI3K pathway, which plays essential roles in tumor progression. Although an association between SECTM1 and STS has not yet been identified, our study provides insight into the tumor-associated immune mechanisms of STS, and the overexpression of SECTM1 may be important in STS development. Several genes were included in the OS-prognostic signature and the PFS-prognostic signature. All of them were confirmed to be relevant to the pathogenesis and prognosis of sarcoma. For example, Chung et al. [58] developed a polyclonal antibody against ILK and found that ILK expression was observed in Ewing’s sarcoma (ES, 100%), which indicated that ILK may be a specific and sensitive immunohistochemical marker for ES. In addition, Et-1 was shown to increase the expression of VEGF and angiogenesis via ILK, resulting in the migration and tube formation of chondrosarcoma cells [59]. Moreover, the expression of RAF1, a part of the MAPK/ERK pathway, is related to cell proliferation in osteosarcoma [60]. Hicks et al. [61] revealed a novel MTAP-RAF1 fusion in a 51-year-old sarcoma patient. Metalloproteases-9 (MMP9) is secreted by metastatic cells and was shown to be highly associated with ES invasion and metastasis [62], and the expression and distribution of MMP9 are related to the occurrence of metastasis and clinical outcomes in STS patients [63]. Hence, the regulation of these IRGs may represent a significant breakthrough in tumor immunotherapy, as the immune system plays a crucial role in the occurrence and progression of cancer [12]. The potential mechanisms by which these genes are involved in sarcoma require further clarification through experimental research.

We also constructed two comprehensive nomograms with satisfactory AUCs (OS: 0.832–0.926, PFS: 0.776–0.874) based on independent variables to assess the deterioration and survival of patients. To date, numerous studies have developed signatures based on sequencing data to stratify sarcoma patients, including CINSARC [64], alternative splicing events [65], relapse-related genes [66], and lncRNA [67]. However, none of them has been applied in clinical practice. Moreover, the use of specific biomarkers with a limited sample size to generate a risk score, which easily leads to overfitting, has no link to clinical reality. However, our prognostic models combining DEGs (which have a significant association with OS and PFS) and IRGs (the expression of which is strongly connected with immune infiltration and tumor progression) is essentially more generic than normal signatures. Therefore, our two nomograms based on the DEIRG-based prognostic signatures and clinicopathologic data can improve the assessment of risk in STS patients.

Some limitations of this study should be noted. First, the training and validation sets came from a retrospective study, which has an inherent bias, and some valuable variables were unavailable. Second, although the signatures were validated by an independent validation set, the prognostic ability in other ethnic groups remains unclear. Finally, the present study is a bioinformatic analysis, and the potential functional mechanisms of IRGs were not studied. Hence, further cell and animal studies should be performed to clearly elucidate the role of IRGs in STS.

## Conclusion

In summary, four IRG-based clusters and two IRG-based signatures were constructed from sarcoma patient data. The clusters showed significant associations with the TME, immune checkpoints, and immune cells. In addition, two TF-IRG regulatory networks were generated, and one key regulatory network was identified and verified. Future in-depth studies should be performed to explore the precise role of IRGs in sarcoma.

## Supplementary information

**Additional file 1:** The entire set of 2498 immune-related genes.

**Additional file 2:** The list of 57 overall survival-related differentially expressed immune-related genes.

**Additional file 3:** The list of 33 progression free survival-related differentially expressed immune-related genes.

**Additional file 4:** The result of LASSO analysis.

**Additional file 5:** The performance of prognostic models in the total cohort.

**Additional file 6:** Subgroup analyses of prognostic signatures.

## Data Availability

The data of this study are from TCGA, GTEx, and GEO database.

## Abbreviations

IRGs: Immune-related genes
STS: Soft tissue sarcoma
TCGA: The Cancer Genome Atlas
GTEx: Genotype-Tissue Expression
DEG: Differentially expressed genes
DEIRGs: Differentially expressed immune-related gene
TME: Tumor microenvironment
OS: Overall survival
PFS: Progression free survival
TF: Transcription factor
FDR: False discovery rate
FC: Fold change
GO: Gene ontology
KEGG: Kyoto encyclopedia of genes and genomes
LASSO: Least absolute shrinkage and selection operator
ROC: Receiver operating characteristic
AUC: Area under the curve
K-M: Kaplan-Meier
C-index: Concordance index
DETF: Differently expressed TF
ES: Ewing’s sarcoma
BP: Biological process
CC: Cellular component
MF: Molecular function
GEO: Gene Expression Omnibus

## References

[1] Toro JR, Travis LB, Wu HJ, Zhu K, Fletcher CDM, Devesa SS (2006). Incidence patterns of soft tissue sarcomas, regardless of primary site, in the surveillance, epidemiology and end results program, 1978–2001: an analysis of 26,758 cases. Int J Cancer.

[2] Jo VY, Fletcher CDM: WHO classification of soft tissue tumours: an update based on the 2013 (4th) edition. Pathology 2014, 46 10.1097/PAT.0000000000000050 24378391

[3] Wibmer C, Leithner A, Zielonke N, Sperl M, Windhager R (2010). Increasing incidence rates of soft tissue sarcomas? A population-based epidemiologic study and literature review. Ann Oncol.

[4] Hung GY, Horng JL, Lee YS, Yen HJ, Chen CC, Lee CY (2014). Cancer incidence patterns among children and adolescents in Taiwan from 1995 to 2009: a population-based study. Cancer.

[5] Kim HS, Nam CM, Jang S-Y, Choi SK, Han M, Kim S, Moneta MV, Lee SY, Cho JM, Novick D, Rha SY (2019). Characteristics and treatment patterns of patients with advanced soft tissue sarcoma in iKorea. Cancer Research And Treatment: Official Journal of Korean Cancer Association.

[6] Kollár A, Rothermundt C, Klenke F, Bode B, Baumhoer D, Arndt V, Feller A (2019). Incidence, mortality, and survival trends of soft tissue and bone sarcoma in Switzerland between 1996 and 2015. Cancer Epidemiol.

[7] Bessen T, Caughey GE, Shakib S, Potter JA, Reid J, Farshid G, Roder D, Neuhaus SJ (2019). A population-based study of soft tissue sarcoma incidence and survival in Australia: an analysis of 26,970 cases. Cancer Epidemiol.

[8] Italiano A, Mathoulin-Pelissier S, Cesne AL, Terrier P, Bonvalot S, Collin F, Michels J-J, Blay J-Y, Coindre J-M, Bui B (2011). Trends in survival for patients with metastatic soft-tissue sarcoma. Cancer.

[9] Min L, Garbutt C, Tu C, Hornicek F, Duan Z: Potentials of Long Noncoding RNAs (LncRNAs) in Sarcoma: From Biomarkers to Therapeutic Targets. International journal of molecular sciences 2017. 18. 10.3390/ijms18040731 PMC5412317 28353666

[10] Kohama I, Kosaka N, Chikuda H, Ochiya T: An Insight into the Roles of MicroRNAs and Exosomes in Sarcoma. Cancers 2019. 11. 10.3390/cancers11030428 PMC6468388 30917542

[11] Liu J, Li R, Liao X, Hu B, Yu J (2019). Comprehensive investigation of the clinical significance and molecular mechanisms of plasmacytoma variant translocation 1 in sarcoma using genome-wide RNA sequencing data. J Cancer.

[12] Gentles AJ, Newman AM, Liu CL, Bratman SV, Feng W, Kim D, Nair VS, Xu Y, Khuong A, Hoang CD (2015). The prognostic landscape of genes and infiltrating immune cells across human cancers. Nat Med.

[13] Shi X, Li R, Dong X, Chen AM, Liu X, Lu D, Feng S, Wang H, Cai K (2020). IRGS: an immune-related gene classifier for lung adenocarcinoma prognosis. J Trans Med.

[14] Zhang M, Zhu K, Pu H, Wang Z, Zhao H, Zhang J, Wang Y: An Immune-Related Signature Predicts Survival in Patients With Lung Adenocarcinoma. Frontiers in Oncology 2019. 9. 10.3389/fonc.2019.01314 PMC6914845 31921619

[15] Shen S, Wang G, Zhang R, Zhao Y, Yu H, Wei Y, Chen F (2019). Development and validation of an immune gene-set based Prognostic signature in ovarian cancer. EBioMedicine.

[16] He Y, Dang Q, Li J, Zhang Q, Yu X, Xue M, Guo W (2020). Prediction of hepatocellular carcinoma prognosis based on expression of an immune-related gene set. Aging.

[17] She Y, Kong X, Ge Y, Yin P, Liu Z, Chen J, Gao F, Fang S (2020). Immune-related gene signature for predicting the prognosis of head and neck squamous cell carcinoma. Cancer Cell Int.

[18] Lin P, Guo Y, Shi L, Li X, Yang H, He Y, Li Q, Dang Y, Wei Kl, Chen G. Development of a prognostic index based on an immunogenomic landscape analysis of papillary thyroid cancer. Aging. 2019. 11:480 10.18632/aging.101754 PMC6366981 30661062

[19] Qiu H, Hu X, He C, Yu B, Li Y, Li J: Identification and Validation of an Individualized Prognostic Signature of Bladder Cancer Based on Seven Immune Related Genes. Frontiers in Genetics. 2020. 11. 10.3389/fgene.2020.00012 PMC7013035 32117435

[20] Shen C, Liu J, Wang J, Zhong X, Dong D, Yang X, Wang Y (2020). Development and validation of a prognostic immune-associated gene signature in clear cell renal cell carcinoma. Int Immunopharmacol.

[21] Goldman M, Craft B, Swatloski T, Cline M, Morozova O, Diekhans M, Haussler D, Zhu J (2015). The UCSC Cancer Genomics Browser: update 2015. Nucleic Acids Res.

[22] Gao J, Aksoy BA, Dogrusoz U, Dresdner G, Gross B, Sumer SO, Sun Y, Jacobsen A, Sinha R, Larsson E, et al: Integrative analysis of complex cancer genomics and clinical profiles using the cBioPortal. Sci Signal 2013, 6:pl1. 10.1126/scisignal.2004088 PMC4160307 23550210

[23] Bhattacharya S, Andorf S, Gomes L, Dunn P, Schaefer H, Pontius J, Berger P, Desborough V, Smith T, Campbell J (2014). ImmPort: disseminating data to the public for the future of immunology. Immunol Res.

[24] Yu G, Wang L-G, Han Y, He Q-Y (2012). clusterProfiler: an R package for comparing biological themes among gene clusters. OMICS.

[25] Wilkerson MD, Hayes DN (2010). ConsensusClusterPlus: a class discovery tool with confidence assessments and item tracking. Bioinformatics.

[26] Engebretsen S, Bohlin J (2019). Statistical predictions with glmnet. Clin Epigenetics.

[27] Camp RL, Dolled-Filhart M, Rimm DL (2004). X-tile: a new bio-informatics tool for biomarker assessment and outcome-based cut-point optimization. Clin Cancer Res.

[28] Hu Y-X, Zheng M-J, Zhang W-C, Li X, Lin B (2019). Systematic profiling of alternative splicing signature reveals prognostic predictor for cervical cancer. J Trans Med.

[29] Shannon P, Markiel A, Ozier O, Baliga NS, Wang JT, Ramage D, Amin N, Schwikowski B, Ideker T (2003). Cytoscape: a software environment for integrated models of biomolecular interaction networks. Genome Res.

[30] Hoffman RM, Singh AS, Eilber FC (2017). Potential of immunotherapy for sarcoma. Cancer.

[31] Sharma P, Allison JP (2015). The future of immune checkpoint therapy. Science.

[32] Petitprez F, de Reyniès A, Keung EZ, Chen TWW, Sun CM, Calderaro J, Jeng YM, Hsiao LP, Lacroix L, Bougoüin A, et al: B cells are associated with survival and immunotherapy response in sarcoma. Nature 2020, 577:556-560. 10.1038/s41586-019-1906-8 31942077

[33] Oliva M, Spreafico A, Taberna M, Alemany L, Coburn B, Mesia R, Siu LL (2019). Immune biomarkers of response to immune-checkpoint inhibitors in head and neck squamous cell carcinoma. Annals Oncol.

[34] Gibney GT, Weiner LM, Atkins MB (2016). Predictive biomarkers for checkpoint inhibitor-based immunotherapy. Lancet Oncol.

[35] Ahn M-J, Sun J-M, Lee S-H, Ahn JS, Park K (2017). EGFR TKI combination with immunotherapy in non-small cell lung cancer. Expert Opinion Drug Safety.

[36] Shen X, Shen P, Yang Q, Yin Q, Wang F, Cong H, Wang X, Ju S (2019). Knockdown of long non-coding RNA PCAT-1 inhibits myeloma cell growth and drug resistance via p38 and JNK MAPK pathways. J Cancer.

[37] Li B, Cui Y, Diehn M, Li R (2017). Development and validation of an individualized immune prognostic signature in early-stage nonsquamous non-small cell lung cancer. JAMA Oncol.

[38] Wang Z, Song Q, Yang Z, Chen J, Shang J, Ju W (2019). Construction of immune-related risk signature for renal papillary cell carcinoma. Cancer Med.

[39] Stefanidakis M, Koivunen E (2006). Cell-surface association between matrix metalloproteinases and integrins: role of the complexes in leukocyte migration and cancer progression. Blood.

[40] Fabre J, Giustiniani J, Garbar C, Antonicelli F, Merrouche Y, Bensussan A, Bagot M, Al-Dacak R: Targeting the Tumor Microenvironment: The Protumor Effects of IL-17 Related to Cancer Type. International journal of molecular sciences 2016, 17. 10.3390/ijms17091433 PMC5037712 27589729

[41] Coussens LM, Werb Z (2001). Inflammatory cells and cancer: think different!. J Exp Med.

[42] Fugl A, Andersen CL (2019). Epstein-Barr virus and its association with disease - a review of relevance to general practice. BMC family practice.

[43] Teitell MA (2005). The TCL1 family of oncoproteins: co-activators of transformation. Nat Rev Cancer.

[44] Renaude E, Kroemer M, Loyon R, Binda D, Borg C, Guittaut M, Hervouet E, Peixoto P: The Fate of Th17 Cells is Shaped by Epigenetic Modifications and Remodeled by the Tumor Microenvironment. International journal of molecular sciences 2020, 21. 10.3390/ijms21051673 PMC7084267 32121394

[45] T B, V D, LEL H, AM D: Immunotherapy: From Advanced NSCLC to Early Stages, an Evolving Concept. Frontiers in medicine 2020, 7:90. 10.3389/fmed.2020.00090 PMC7105823 32266275

[46] H Y, K W, T W, M L, B L, S L, L Y: The Combination Options and Predictive Biomarkers of PD-1/PD-L1 Inhibitors in Esophageal Cancer. Frontiers in oncology 2020, 10:300. 10.3389/fonc.2020.00300 PMC7066251 32195194

[47] EX C, DJ J, JM L, HF K, SR B, F C, CE A, JR G, P K, M H, et al: Effect of Combined Immune Checkpoint Inhibition vs Best Supportive Care Alone in Patients With Advanced Colorectal Cancer: The Canadian Cancer Trials Group CO.26 Study. JAMA oncology 2020. 10.1001/jamaoncol.2020.0910 PMC7206536 32379280

[48] R H-B, E H, S K, S M, S F, G E, A Y, M O, N C, T P, et al: Immunotherapy Potentiates the Effect of Chemotherapy in Metastatic Melanoma-A Retrospective Study. Frontiers in oncology 2020, 10:70. 10.3389/fonc.2020.00070 PMC7033746 32117727

[49] AW S, HL K, B C, JM M, K M, D M, J C, J N, PK B, L D, et al: High-Dose Ipilimumab and High-Dose Interleukin-2 for Patients With Advanced Melanoma. Frontiers in oncology 2019, 9:1483. 10.3389/fonc.2019.01483 PMC6965158 31998643

[50] C DA, M B, C C, S R, BA F, G I, G M, M C, CD P, L P, et al: Biomarkers of Prognosis and Efficacy of Anti-angiogenic Therapy in Metastatic Clear Cell Renal Cancer. Frontiers in oncology 2019, 9:1400. 10.3389/fonc.2019.01400 PMC6917607 31921657

[51] Boxberg M, Steiger K, Lenze U, Rechl H, von Eisenhart-Rothe R, Wörtler K, Weichert W, Langer R, Specht K (2017). PD-L1 and PD-1 and characterization of tumor-infiltrating lymphocytes in high grade sarcomas of soft tissue - prognostic implications and rationale for immunotherapy. Oncoimmunology.

[52] Orth MF, Buecklein VL, Kampmann E, Subklewe M, Noessner E, Cidre-Aranaz F, Romero-Pérez L, Wehweck FS, Lindner L, Issels R (2020). A comparative view on the expression patterns of PD-L1 and PD-1 in soft tissue sarcomas.

[53] Kawamoto K, Miyoshi H, Suzuki T, Kiyasu J, Yokoyama S, Sasaki Y, Sone H, Seto M, Takizawa J, Ohshima K (2018). Expression of programmed death ligand 1 is associated with poor prognosis in myeloid sarcoma patients. Hematol Oncol.

[54] Iimuro S, Shindo T, Moriyama N, Amaki T, Niu P, Takeda N, Iwata H, Zhang Y, Ebihara A, Nagai R (2004). Angiogenic effects of adrenomedullin in ischemia and tumor growth. Circ Res.

[55] Tanaka M, Koyama T, Sakurai T, Kamiyoshi A, Ichikawa-Shindo Y, Kawate H, Liu T, Xian X, Imai A, Zhai L (2016). The endothelial adrenomedullin-RAMP2 system regulates vascular integrity and suppresses tumour metastasis. Cardiovasc Res.

[56] Tan A, Porcher R, Crequit P, Ravaud P, Dechartres A: Differences in Treatment Effect Size Between Overall Survival and Progression-Free Survival in Immunotherapy Trials: A Meta-Epidemiologic Study of Trials With Results Posted at ClinicalTrials.gov. Journal of Clinical Oncology Official Journal of the American Society of Clinical Oncology 2017, 35:JCO2016712109. 10.1200/JCO.2016.71.2109 28375786

[57] Wang T, Ge Y, Xiao M, Lopez-Coral A, Li L, Roesch A, Huang C, Alexander P, Vogt T, Xu X (2014). SECTM1 produced by tumor cells attracts human monocytes via CD7-mediated activation of the PI3K pathway. J Invest Dermatol.

[58] Chung DH, Lee JI, Kook MC, Kim JR, Kim SH, Choi EY, Park SH, Song HG (1998). ILK (beta1-integrin-linked protein kinase): a novel immunohistochemical marker for Ewing’s sarcoma and primitive neuroectodermal tumour. Virchows Archiv: an international journal of pathology.

[59] Wu MH, Huang CY, Lin JA, Wang SW, Peng CY, Cheng HC, Tang CH (2014). Endothelin-1 promotes vascular endothelial growth factor-dependent angiogenesis in human chondrosarcoma cells. Oncogene.

[60] Sasaki K, Hitora T, Nakamura O, Kono R, Yamamoto T (2011). The role of MAPK pathway in bone and soft tissue tumors. Anticancer Res.

[61] Hicks JK, Henderson-Jackson E, Duggan J, Joyce DM, Brohl AS (2018). Identification of a novel MTAP-RAF1 fusion in a soft tissue sarcoma. Diagnostic Pathol.

[62] Sancéau J, Truchet S, Bauvois B (2003). Matrix metalloproteinase-9 silencing by RNA interference triggers the migratory-adhesive switch in Ewing’s sarcoma cells. J Biol Chem.

[63] Benassi MS, Gamberi G, Magagnoli G, Molendini L, Ragazzini P, Merli M, Chiesa F, Balladelli A, Manfrini M, Bertoni F (2001). Metalloproteinase expression and prognosis in soft tissue sarcomas. Annals Oncol.

[64] Chibon F, Lesluyes T, Valentin T, Le Guellec S (2019). CINSARC signature as a prognostic marker for clinical outcome in sarcomas and beyond. Genes Chromosom Cancer.

[65] Yang X, Huang W-T, He R-Q, Ma J, Lin P, Xie Z-C, Ma F-C, Chen G (2019). Determining the prognostic significance of alternative splicing events in soft tissue sarcoma using data from The Cancer Genome Atlas. J Trans Med.

[66] Hu Q, Zhou S, Hu X, Zhang H, Huang S, Wang Y (2020). Systematic screening identifies a 2-gene signature as a high-potential prognostic marker of undifferentiated pleomorphic sarcoma/myxofibrosarcoma. J Cell Mol Med.

[67] He R-q, Wei Q-j, Tang R-x, Chen W-j (2017). Yang X, Peng Z-g, Hu X-h, Ma J, Chen G: prediction of clinical outcome and survival in soft-tissue sarcoma using a ten-lncRNA signature. Oncotarget.

