# Supplementary figures and images for: Comprehensive profiling of immune-related genes in soft tissue sarcoma patients
Source: J Transl Med. 2020 Sep 1;18:337. doi: 10.1186/s12967-020-02512-8 (PMC7465445; doi:10.1186/s12967-020-02512-8)

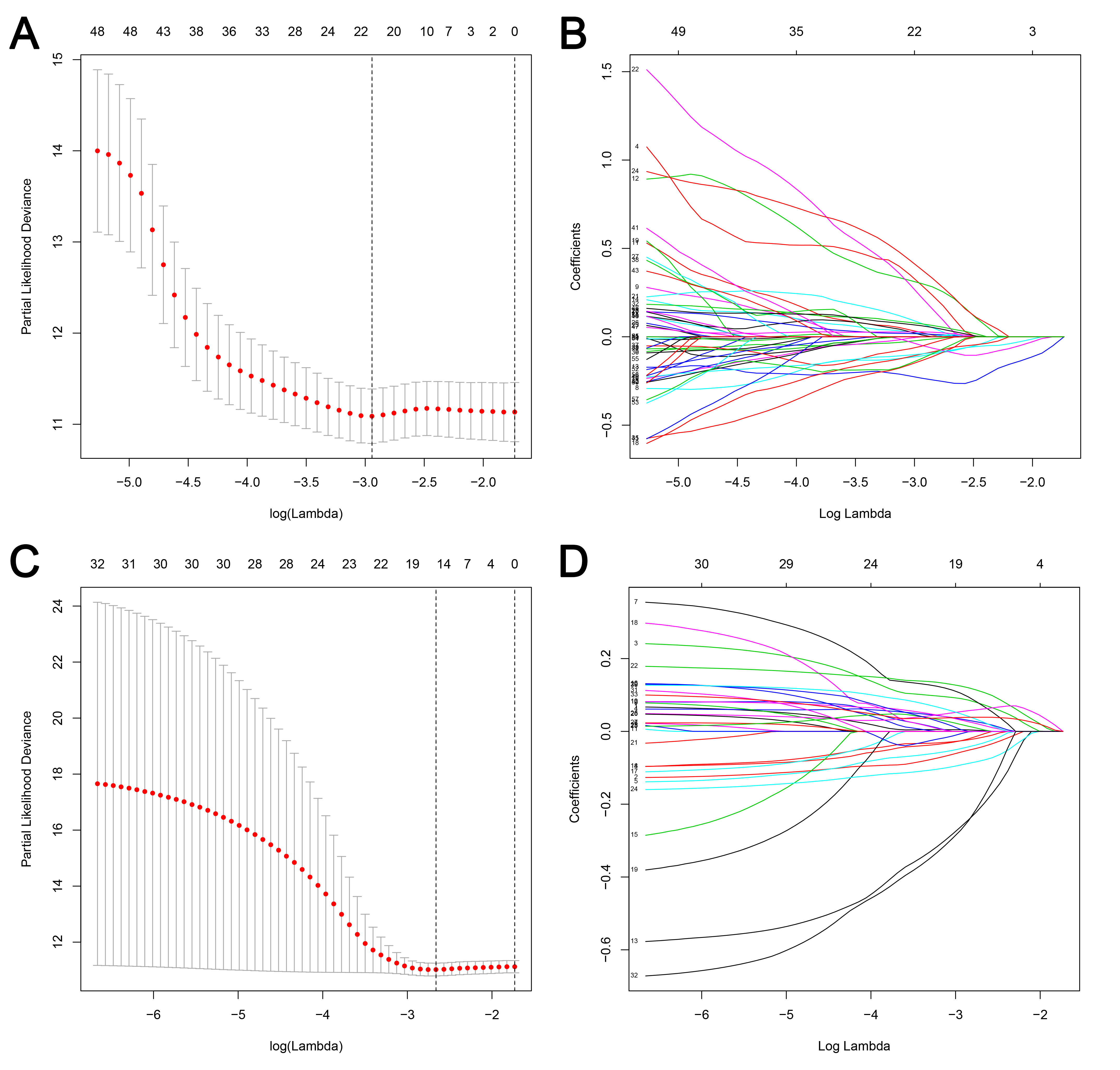

Supplement: Supplementary file 4 — Additional file 4: The result of LASSO analysis. [file 12967_2020_2512_MOESM4_ESM.tif]

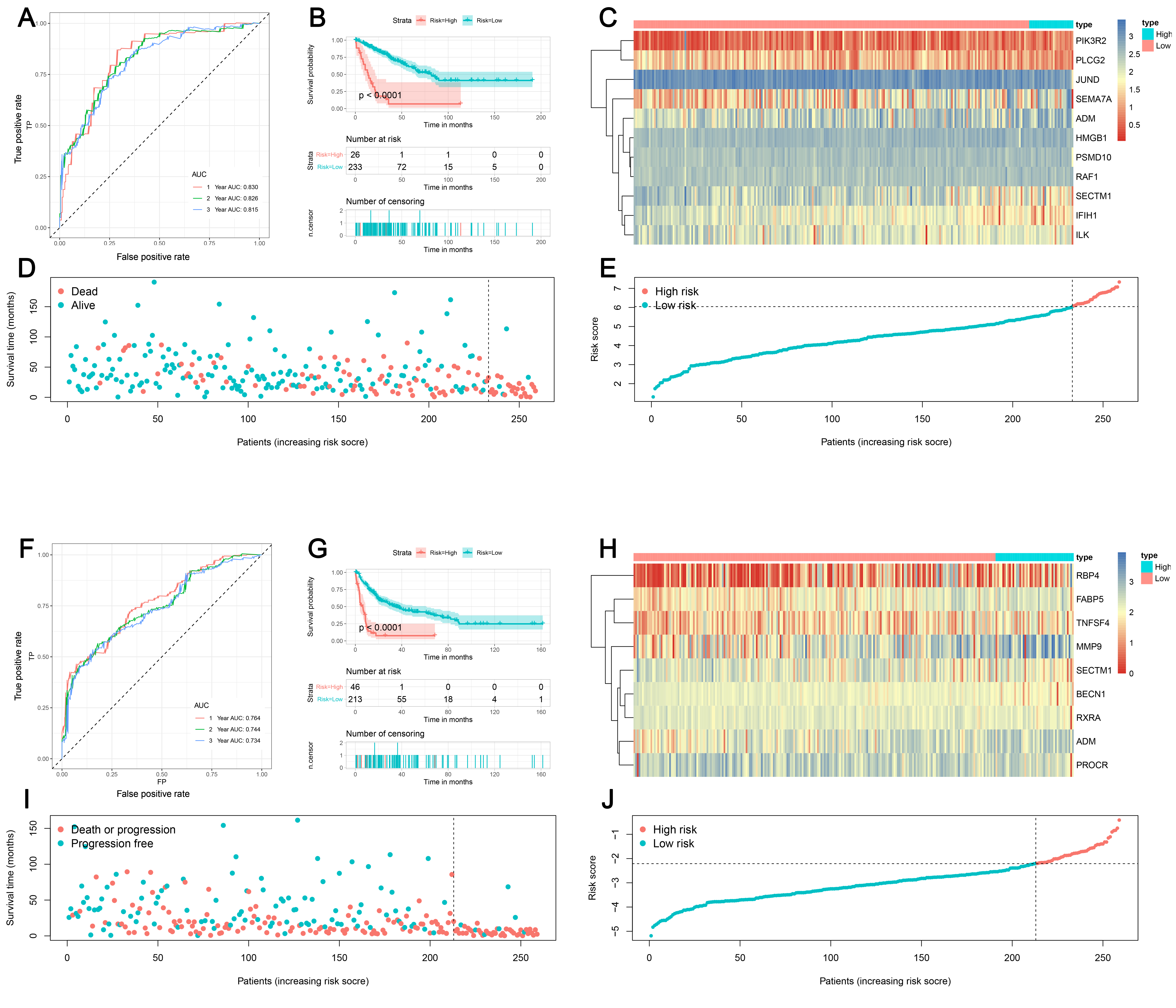

Supplement: Supplementary file 5 — Additional file 5: The performance of prognostic models in the total cohort. [file 12967_2020_2512_MOESM5_ESM.tif]

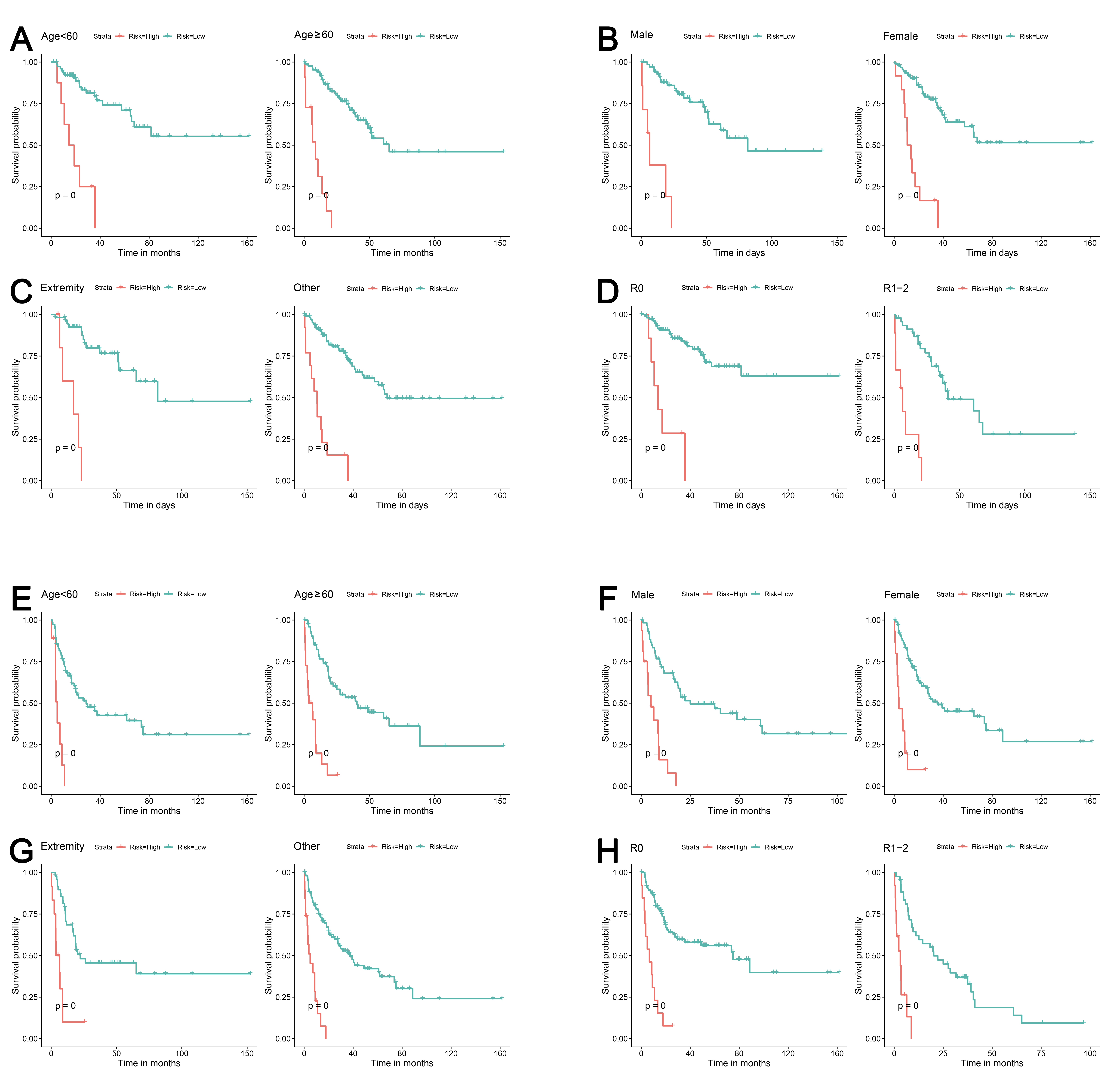

Supplement: Supplementary file 6 — Additional file 6: Subgroup analyses of prognostic signatures. [file 12967_2020_2512_MOESM6_ESM.tif]
